# Ventilation practices in burn patients—an international prospective observational cohort study

**DOI:** 10.1093/burnst/tkab034

**Published:** 2021-12-16

**Authors:** Gerie J Glas, Janneke Horn, Markus W Hollmann, Benedikt Preckel, Kirsten Colpaert, Manu Malbrain, Ary Serpa Neto, Karim Asehnoune, Marcello Gamma de Abreu, Ignacio Martin-Loeches, Paolo Pelosi, Folke Sjöberg, Jan M Binnekade, Berry Cleffken, Nicole P Juffermans, Paul Knape, Bert G Loef, David P Mackie, Perenlei Enkhbaatar, Nadia Depetris, Anders Perner, Eva Herrero, Lucia Cachafeiro, Marc Jeschke, Jeffrey Lipman, Matthieu Legrand, Johannes Horter, Athina Lavrentieva, Alex Kazemi, Anne Berit Guttormsen, Frederik Huss, Mark Kol, Helen Wong, Therese Starr, Luc De Crop, Wilson de Oliveira Filho, João Manoel Silva Junior, Cintia M C Grion, Marjorie Burnett, Frederik Mondrup, Francois Ravat, Mathieu Fontaine, Renan Le Floch, Mathieu Jeanne, Morgane Bacus, Maïté Chaussard, Marcus Lehnhardt, Bassem Daniel Mikhail, Jochen Gille, Aidan Sharkey, Nicole Trommel, Auke C Reidinga, Nadine Vieleers, Anna Tilsley, Henning Onarheim, Maria Teresa Bouza, Alexander Agrifoglio, Filip Fredén, Tina Palmieri, Lynda E Painting, Marcus J Schultz, LAMiNAR investigators

**Affiliations:** Academic Medical Center, University of Amsterdam, Amsterdam, AZ 1105, The Netherlands; Academic Medical Center, University of Amsterdam, Amsterdam, AZ 1105, The Netherlands; Academic Medical Center, University of Amsterdam, Amsterdam, AZ 1105, The Netherlands; Academic Medical Center, University of Amsterdam, Amsterdam, AZ 1105, The Netherlands; Department of Anaesthesia and Intensive Therapy Medical University of Lublin Aleje Racklawickie 1 – 20-059 Lublin – Poland; AZ JAN PALFIJN GENT Watersportlaan 5 – 9000 Gent – Belgium; Department of Anaesthesia and Intensive Therapy Medical University of Lublin Aleje Racklawickie 1 – 20-059 Lublin – Poland; ABC Medical School, São Paulo, Bangú, SP 5001, Brazil; Australian and New Zealand Intensive Care Research Centre. Monash University, Melbourne, VIC 3004, Australia; GH St-Louis- Lariboisière, APHP, Paris 75010, France; Service d'Anesthésie Réanimation Chirurgicale, Nantes 44093, France; University Hospital Carl Gustav Carus, Dresden 01307, Germany; St James University Hospital, Dublin D08 NHY1, Ireland; University of Genoa, Genoa, GE 16128, Italy; Linköping University Hospital, Linköping 581 85, Sweden; Academic Medical Center, University of Amsterdam, Amsterdam, AZ 1105, The Netherlands; Maasstad Hospital, Rotterdam, DZ 3079, The Netherlands; Academic Medical Center, University of Amsterdam, Amsterdam, AZ 1105, The Netherlands; Red Cross Hospital, Beverwijk, LE 1942, The Netherlands; Martini Hospital, Groningen, NT 9728, The Netherlands; Red Cross Hospital, Beverwijk, LE 1942, The Netherlands; University of Texas Medical Branch, Galveston, TX 77555, USA; Turin CTO Burn Center, Turin, TO 10126, Italy; Rigshospitalet, Copenhagen 2100, Denmark; La Paz University Hospital, Madrid 28046, Spain; La Paz University Hospital, Madrid 28046, Spain; Ross Tilley Burn Centre, Sunnybrook Health Sciences Centre, Toronto M4N 3M5, Canada; Royal Brisbane and Women’s Hospital, Queensland University, Herston, QLD 4029, Australia; GH St-Louis- Lariboisière, APHP, Paris 75010, France; Hopital Roger Salengro, CHRU Lille, Lille 59037, France; BG Klinik Ludwigshafen, Ludwigshafen 67071, Germany; Papanikoalou Hospital, Thessaloniki 546 21, Greece; Middlemore Hospital, Otahuhu, Auckland 2025, New Zealand; Haukeland University Hospital, Bergen 5021, Norway; Uppsala University Hospital, Uppsala 751 85, Sweden; Concord Repatriation General Hospital NSW, University of Sydney, Concord 2139, Australia; Concord Repatriation General Hospital NSW, University of Sydney, Concord 2139, Australia; Royal Brisbane and Women’s Hospital, Queensland University, Herston, QLD 4029, Australia; Department of Anaesthesia and Intensive Therapy Medical University of Lublin Aleje Racklawickie 1 – 20-059 Lublin – Poland; Ziekenhuis Netwerk Antwerpen–Stuivenberg, Antwerpen 2060, Belgium; Hospital e Pronto Socorro 28 de Agosto, Manaus 69057-000, Brazil; Universidade de Sao Paulo, Sao Paulo 01246-903, Brazil; Universidade Estadual de Londrina, Londrina 86057-970, Brazil; Rigshospitalet, Copenhagen 2100, Denmark; Sunnybrook Health Sciences Centre, Toronto M4N 3M5, Canada; Sunnybrook Health Sciences Centre, Toronto M4N 3M5, Canada; CHU Lyon St Luc, Lyon 69007, France; CHU Nantes Service dánesthesie reanimation chirugicale, Nantes 44093, France; CHU Nantes Service dánesthesie reanimation chirugicale, Nantes 44093, France; Hopital Roger Salengro, CHRU Lille, Lille 59037, France; Saint-Louis Hospital, Paris 75010, France; Saint-Louis Hospital, Paris 75010, France; BG University Hospital Bergmannsheil, Bochum 44789, Germany; St James University Hospital, Dublin D08 NHY1, Ireland; Maasstad Hospital, Rotterdam, DZ 3079, The Netherlands; Martini Hospital, Groningen, NT 9728, The Netherlands; Red Cross Hospital, Beverwijk, LE 1942, The Netherlands; Middlemore Hospital, Otahuhu, Auckland 2025, New Zealand; Haukeland University Hospital, Bergen 5021, Norway; St George Leipzig, Leipzig 04129, Germany; La Paz University Hospital, Madrid 28046, Spain; Uppsala University Hospital, Uppsala 751 85, Sweden; St. James Hospital, Dublin D08 NHY1, Ireland; St. James Hospital, Dublin D08 NHY1, Ireland; Academic Medical Center, University of Amsterdam, Amsterdam, AZ 1105, The Netherlands

**Keywords:** Mechanical ventilation, Inhalation trauma, Lung-protective, Critical care

## Abstract

**Background:**

It is unknown whether lung-protective ventilation is applied in burn patients and whether they benefit from it. This study aimed to determine ventilation practices in burn intensive care units (ICUs) and investigate the association between lung-protective ventilation and the number of ventilator-free days and alive at day 28 (VFD-28).

**Methods:**

This is an international prospective observational cohort study including adult burn patients requiring mechanical ventilation. Low tidal volume (*V*_T_) was defined as *V*_T_ ≤ 8 mL/kg predicted body weight (PBW). Levels of positive end-expiratory pressure (PEEP) and maximum airway pressures were collected. The association between *V*_T_ and VFD-28 was analyzed using a competing risk model. Ventilation settings were presented for all patients, focusing on the first day of ventilation. We also compared ventilation settings between patients with and without inhalation trauma.

**Results:**

A total of 160 patients from 28 ICUs in 16 countries were included. Low *V*_T_ was used in 74% of patients, median *V*_T_ size was 7.3 [interquartile range (IQR) 6.2–8.3] mL/kg PBW and did not differ between patients with and without inhalation trauma (*p* = 0.58). Median VFD-28 was 17 (IQR 0–26), without a difference between ventilation with low or high *V*_T_ (*p* = 0.98). All patients were ventilated with PEEP levels ≥5 cmH_2_O; 80% of patients had maximum airway pressures <30 cmH_2_O.

**Conclusion:**

In this international cohort study we found that lung-protective ventilation is used in the majority of burn patients, irrespective of the presence of inhalation trauma. Use of low *V*_T_ was not associated with a reduction in VFD-28.

**Trial registration:**

Clinicaltrials.gov NCT02312869. Date of registration: 9 December 2014.

Highlights

- First international prospective observational study investigating mechanical ventilation practices in specialized adult burn intensive care units.
- Lung-protective ventilation is used in the majority of burn patients.
- Use of lung-protective ventilation settings is irrespective of the presence of inhalation trauma.

## Background

Mechanical ventilation (MV) is considered a lifesaving intervention, but it also causes lung injury [1,2]. Ventilator settings important in ‘ventilator-induced lung injury’ (VILI) include tidal volume (*V*_T_) and positive end-expiratory pressure (PEEP). To limit VILI, ‘lung-protective’ MV strategies have become standard care in the general intensive care unit (ICU) [3,4]. *V*_T_ sizes of ≤8 mL/kg predicted body weight (PBW) are preferred [3,4] as patients with and without acute respiratory distress syndrome (ARDS) benefit from low *V*_T_ [1,5–8]. Current guidelines suggest the use of higher PEEP (e.g. >10 cmH_2_O) for patients with moderate to severe ARDS [9,10]. The optimal PEEP for patients without ARDS remains debatable. However, a trend towards the use of moderate PEEP, generally between 5 and 10 cmH_2_O, has been reported [4,11].

Burn patients often suffer from inhalation trauma. Both thermal and inhalation trauma may result in respiratory dysfunction, necessitating MV [12–14]. Whether lung-protective ventilation is applied in burn patients is yet unknown. There are no evidence-based guidelines for MV in burn patients. Furthermore, it is unknown whether this specific population benefits from lung-protective ventilation as data on the association between ventilation practices and clinical outcomes are scant [15].

To determine ventilation practices in burn ICUs worldwide we performed an international prospective observational cohort study entitled ‘Local Assessment of MaNAgement in BuRn Patients’ (LAMiNAR). We expected extensive variability in ventilation practices. The secondary objective was to determine the association between ventilation settings, focusing on *V*_T_ size and levels of PEEP, and duration of ventilation in burn patients, with the number of ventilator-free days and alive at day 28 (VFD-28) as the primary outcome measure.

## Methods

### Design

The LAMiNAR study is a prospective observational international cohort study in specialized burn ICUs. Burn patients were included during a 3-month period per participating center. The study protocol was centrally approved by the Institutional Review Board (IRB) of the Academic Medical Center at the University of Amsterdam, The Netherlands (W14_314#15.0178). Locally, ethical approval was obtained in compliance with the local regulatory requirements. If applicable, written informed consent from individual patients or legal representatives was obtained prior to enrollment. The study was registered at www.clinicaltrials.gov (NCT02312869). National coordinators were appointed in participating countries (see online supplementary appendix, Table 2); they recruited collaborating centers and assisted local coordinators.

### Study population

Consecutive adult (≥18 years) burn patients admitted to a participating burn ICU who needed invasive MV, irrespective of severity of burn injury or presence of inhalation trauma, were eligible for inclusion. There were no exclusion criteria.

### Data collection

Patient characteristics and baseline data were collected on the day of ICU admission: Simplified Acute Physiology Score (SAPS) II, Lung injury scores (LIS) [16] and Sequential Organ Failure Assessment (SOFA) scores [17]; data on etiology and severity of burn injury (e.g. percentage of total body surface area burned (TBSA %); presence of inhalation trauma: not suspected, clinical diagnosis, or bronchoscopically confirmed; severity of inhalation trauma graded as mild (i.e. minor or patchy areas of erythema, carbonaceous deposits in bronchi), moderate (i.e. moderate degree of erythema, carbonaceous deposits, bronchorrhea, or bronchial obstruction) or severe (i.e. severe inflammation with friability, copious carbonaceous deposits, bronchorrhea, obstruction or evidence of mucosal sloughing, necrosis or obliteration) [18]. Data on the timing of bronchoscopy and applied nebulization protocols (e.g. use of nebulized heparin, mucolytics or bronchodilators) were not collected.

For MV parameters, day 0 was defined as the first day of MV in the ICU. Ventilator data (described below) and clinical outcome parameters were collected daily until day seven, death or discharge from ICU, whichever came first. All ventilatory data were single measurements collected at the same time point. Daily data were collected from the morning round if the patient was stable for at least 1 h; i.e. closest to 08:00 am, otherwise data from 1 h earlier or later was collected. Whether a patient was considered stable was left at the discretion of the attending physician.

Clinical outcome parameters included: VFD-28, a VFD was defined as a period of 24 consecutive hours in which the patient was alive and without MV, in hospital and ICU length of stay (LOS) and all-cause mortality. Other outcome parameters included: development of pneumonia (i.e. new or progressive radiographic infiltrate plus at least two of the following: fever >38°C, leukocytosis, leucopenia and/or purulent secretions) [4] or ARDS according to the Berlin definition [19] and development of acute renal failure according to acute kidney injury network criteria [20]. Data on the duration of MV, LOS and mortality (in ICU and hospital) were assessed on days 28 and 90. We also collected data on: PaO_2_/fraction of inspired oxygen (FiO_2_) (mmHg), respiratory rate (breaths per minute), pulmonary compliance (mL/cmH_2_O), minute ventilation and arterial blood gas (P_a_CO_2_, pH, bicarbonate).

Ventilatory data included: *V*_T,_ PEEP, FiO_2_, ventilator mode (e.g. high-frequency ventilation, spontaneous or controlled modes), peak airway pressure (*P*_peak_) and plateau pressure (*P*_plat_) for volume-controlled ventilation; maximum airway pressure (*P*_max_) for pressure-controlled ventilation; and driving pressure [calculated as *P*_plat_ (or equivalent) minus PEEP] [21].

Anonymized patient data were entered into a web-based, password-secured, electronic case record form (Openclinica, Boston, MA, USA).

### Study objectives and parameters

The objective of this study was to determine current ventilation practices in burn patients. The main ventilatory parameters were *V*_T_ and PEEP. Secondary ventilatory parameters included FiO_2_, ventilator mode, *P*_peak_ and *P*_plat_ for volume-controlled ventilation; *P*_max_ for pressure-controlled ventilation; and driving pressure. The main outcome parameter was number of VFD-28.

### Sample size and statistical analysis

We aimed to include 300 patients to enable a multivariate analysis to determine the association between ventilator settings and number of VFD-28. A sample size of 300 patients was required to have a power of 0.80, with a significance level of 0.05, using an estimated effect size of 0.40 [22], while using four independent variables (i.e. *V*_T_, PEEP, FiO_2_ and ventilator mode) in the model. Inclusion in the 3-month period per participating center was much lower than expected. Therefore, we decided to deviate from our originally planned analysis for our secondary objective, and only analyzed the association between one ventilatory parameter (*V*_T_) and the number of VFD-28. We did not analyze the association between levels of PEEP and the number of VFD-28 as only five patients were ventilated with high PEEP levels (i.e. >10 cmH_2_O).

**Table 1:** Patient characteristics and severity of burn injury. Mann–Whitney U or Chi square test. One patient without data on burn etiology and severity. All values given as median (interquartile range) if not stated otherwise. Data from ICU admission day

	**All** *n* = 160	**With inhalation trauma** *n* = 84	**Without inhalation trauma** *n* = 75	***P* value**
Gender, male, *n* (%)	119 (75%)	64 (76%)	55 (73%)	0.82
Age (years)	46 (30–60)	48 (30–60)	44 (29–58)	0.48
Height (cm), *n*	175 (167–180), 150	175 (166–180), 81	174 (167–180), 69	0.58
Weight (kg), *n*	80 (70–90), 158	80 (70–90), 84	80 (72–90), 74	0.68
SAPS II	48 (35–60)	49 (37–62)	43 (35–57)	0.08
LIS	0.75 (0.33–1.33)	1 (0.33–1.5)	0.75 (0.27–1.25)	0.16
SOFA (total)	9 (8–10)	9 (8–11)	8 (7–10)	0.09
Type of burn injury^a^, *n* (%)				
Flames or explosion	137 (86.2%)	75 (89.3%)	62 (82.7%)	
Scalds or steam	5 (3.1%)	2 (2.3%)	3 (4%)	
Contact burns	5 (3.1%)	0	5 (6.7%)	
Other	12 (7.5%)	7 (8.3%)	5 (6.7%)	0.10
TBSA (%)	25 (10–40)	24 (10–40)	25 (13–40)	0.38
Presence of full thickness burn, *n* (%)	97 (60.6%)	50 (59.5%)	47 (62.6%)	0.68

^a^One patient had no data.*LIS* lung injury score at admission, *n* number of patients, *SAPS II* simplified acute physiology score, *SOFA* sequential organ failure assessment, *TBSA* total body surface area of burn

Continuous not normally distributed variables were expressed by medians and interquartile ranges (IQRs). Categorical variables were expressed as *n* (%). Groups were compared with the Mann–Whitney U test. Categorical variables were compared with the Chi–square or Fisher’s exact tests.

*V*_T_ was presented as volume normalized for PBW (mL/kg PBW) [5]. Patients in whom PBW could not be calculated were omitted from the analysis. We used scatterplots to present distributions of *V*_T_  *vs* PEEP, *V*_T_  *vs* respiratory rate, *V*_T_  *vs* FiO_2_ and V_T_  *vs* P_max_. Widely accepted cut-off values of 8 mL/kg PBW for *V*_T_, 14 breaths per min for respiratory rate, 5 cmH_2_O for PEEP, 0.6 for FiO_2_ and 30 cmH_2_O for *P*_max_, were used to form the matrices [4].

In a *post hoc* analysis we evaluated differences in ventilation management between patients with and without inhalation trauma, and analyzed the association between the presence of inhalation trauma and number of VFD-28. Patients were stratified into two groups based on the presence [defined as any suspected (clinical diagnosis) or bronchoscopically confirmed inhalation trauma] or absence of inhalation trauma.

We presented ventilation settings for all patients and compared ventilation management between patients with and without inhalation trauma, focusing on the first day of ventilation (day 1).

The median *V*_T_ on the first day of ventilation was used to determine whether ventilation was ‘lung-protective’ (low *V*_T_): ≤8 mL/kg PBW; *V*_T_ >8 mL/kg PBW was considered as non-protective (high *V*_T_).

The association between (1) *V*_T_ size (*V*_T_ ≤8 *vs*  *V*_T_ >8 mL/kg PBW) and (2) inhalation trauma and the number of VFD-28 was analyzed using a competing risk model with death before extubation as competing risk. Data were presented with cumulative incidence curves. The subdistribution hazard ratio for *V*_T_ and inhalation trauma were calculated using a Cox proportional hazards model, and Schoenfeld residuals were used to test the proportional hazard assumption [23]. Duration of ventilation in survivors was compared using median difference from a quantile regression.

We made no assumptions for missing data and did not adjust for multiplicity across analyses. Patients that were enrolled without subsequent data entry (e.g. no daily data collection and no follow-up data) were excluded from analysis. Statistical significance was considered at *p* < 0.05. All analyses were performed with R v.2·12·0 (http://www.r-project.org).

## Results

### Patients

Patients were enrolled between September 2015 and April 2017. In total, 28 specialized burn ICUs in 16 countries participated (see online supplementary appendix Tables 1 and 2). Patient recruitment was lower than expected. Although we expected to include 300 patients within the 3-month periods, only 170 patients were enrolled, of which 160 patients could be included in the analysis (see online supplementary appendix Figure S1).

Demographic, baseline and etiological characteristics are presented in Table 1. The median percentage of TBSA was 25% (IQR 10–40). Inhalation trauma was clinically suspected in 84 out of 159 patients (52.8%) (1 patient had no data available on the presence of inhalation trauma) and was confirmed by bronchoscopy in 45 of these patients (53.6%). Bronchoscopically confirmed inhalation trauma was graded as mild, moderate or severe in respectively 16, 18 and 11 patients. The number of surgical procedures performed in the first day of mechanical ventilation was similar between patients with and without inhalation trauma and included burn wound excisions, performed in 9 patients, debridement (*n* = 7) and escharotomy (*n* = 4).

### Ventilator settings

**Figure 1.**
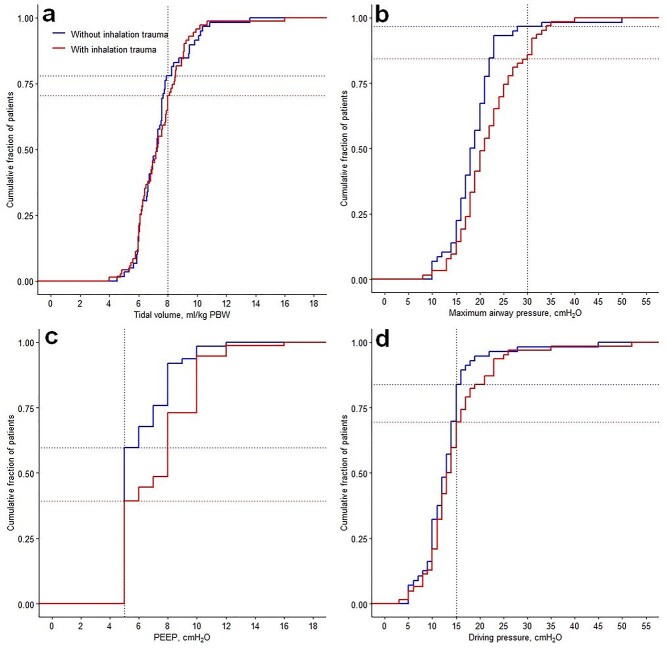
Ventilator settings on the first day of ventilation of patients with and without inhalation trauma. Cumulative frequency distributions from the following parameters measured on the first day of mechanical ventilation: (**a**) *V*_T_, (**b**) maximum airway pressure, (**c**) PEEP, (**d**) driving pressure. Vertical dotted lines: predefined cut-off values for each variable. Horizontal dotted lines: proportion of patients reaching the cut-offs. Driving pressure: plateau (or peak) pressure minus PEEP. *V*_T_ tidal volume, *PEEP* positive end-expiratory pressure, *PBW* predicted body weight

**Figure 2.**
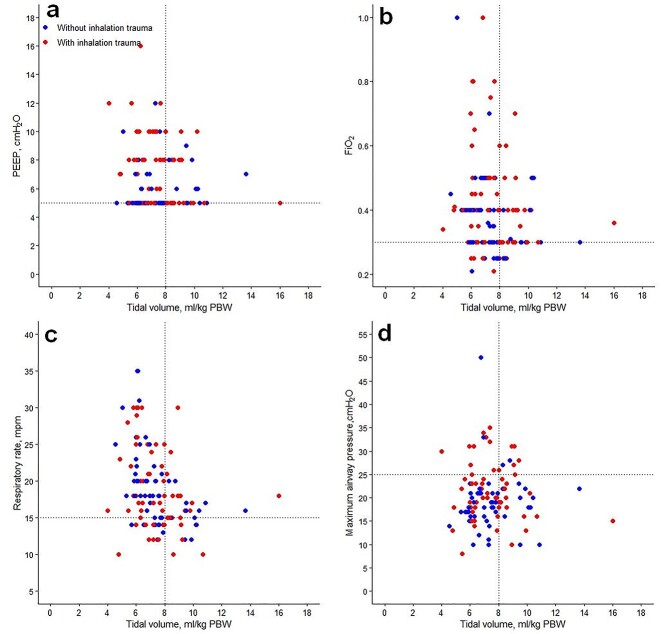
Distribution of ventilatory parameters on the first day of mechanical ventilation. Distribution of positive end-expiratory pressure (PEEP), inspired fraction of oxygen (FiO_2_), respiratory rate and maximum airway pressure *vs* tidal volume (*V*_T_). Dotted lines (horizontal and vertical) represent cut-off values for each variable. (**a**) PEEP, (**b**) FiO_2_, (**c**) respiratory rate, (**d**) maximum airway pressure

Low *V*_T_ were used in 74% of the patients (Table 2, Figures 1 and 2). The median *V*_T_ size was 7.3 (IQR 6.2–8.3) mL/kg PBW for all patients and did not differ significantly between patients with and without inhalation trauma (*p* = 0.58; Table 2). *V*_T_ sizes were similar between patients with clinical and bronchoscopically confirmed diagnosis of inhalation trauma, irrespective of the severity of inhalation trauma (*p* = 0.31). Median *V*_T_ size was significantly higher in patients ventilated with spontaneous [*V*_T_ 8 mL/kg PWB (IQR 7.3–9.5)] compared to controlled ventilation modes [V_T_ 7 mL/kg PWB (IQR 6.1–8), *p* = 0.007].

**Table 2:** Ventilatory parameters on the first day of ventilation. Values given as median (interquartile range) if not stated otherwise. All ventilation and arterial blood gas parameters were collected at the same time point (e.g. 08.00 am if the patient was stable for at least 1 h, otherwise 1 h earlier or later)

	**All**	**With inhalation trauma**	**Without inhalation trauma**	***P* value**
	*N* = 160	*N* = 84	*N* = 75	
*V*_T_ (mL/kg PBW^a^), *n*	7.3 (6.2–8.3), 130	7.3 (6.2–8.5), 71	7.3 (6.2–7.8), 59	0.58
≤8, *n*	96	50	46	
>8, *n*	34	21	13	
Absolute *V*_T_ (mL), *n*/*N*	500 (430–584), 137/160	500 (440–600), 73/84	480 (420–560), 63/75	0.33
Controlled mode, *n*	7 (6.1–8), 107	7.1 (6.1–8.2), 59	7 (6.1–7.8), 48	0.58
Spontaneous mode, *n*	8 (7.3–9.5), 22	8.3 (7.5–9.2), 12	7.6 (7–9.5), 10	0.82
PEEP (cmH_2_O), *n*/*N*	6 (5–8), 135	8 (5–10), 74	5 (5–8), 62	0.004
5, *n*	66	30	37	
6–10, *n*	64	40	24	
>10, *n*	5	4	1	
FiO_2_, *n*	0.35 (0.3–0.4), 155	0.39 (0.3–0.5), 82	0.35 (0.3–0.4), 73	0.36
Peak pressure (cmH_2_O), *n*	23 (19–31), 67	31 (23–35), 29	20 (17–25), 38	<0.001
*P*_plat_ (cmH_2_O), *n*	18 (16–23), 40	21 (18–24), 20	17 (15–23), 20	0.12
*P*_max_ (cmH_2_O), *n*	20 (17–24), 59	20 (17–25), 37	20 (17–22), 22	0.46
Maximum airway pressure^b^, *n*	21 (18–30), 121	24 (19–31), 64	20 (17–24), 58	0.007
Driving pressure^c^ (cmH_2_O), *n*	13 (11–17), 118	14 (11–18), 62	13 (10–16), 56	0.55
Other parameters				
PaO_2_/FiO_2_ (mmHg)	288 (186–402)	275 (172–358)	320 (229–415)	0.05
*n* (%)	154 (96)	73 (90)	6(85)	
Respiratory rate (breaths per minute), *n*	18 (15–21), 138	18 (15–22), 74	18 (15–20), 63	0.71
Compliance (mL/cm H_2_O)^d^, *n*	35.6 (21.2–50), 92	34.3 (20.4–48.8), 61	36.2 (21.7–51.8), 31	0.59
Minute ventilation (L/min), *n*	8.8 (7.6–10.8), 130	8.8 (7.5–11), 71	8.8 (7.6–10), 59	0.42
PaCO_2_ (mmHg), *n*	41 (36–45), 142	41 (36–47), 73	41 (36–44), 68	0.46
Arterial blood pH, *n*	7.39 (7.32–7.43), 142	7.38 (7.31–7.43), 73	7.40 (7.34–7.43), 68	0.31
HCO_2_ (mEq/L), *n*	23 (21–26), 142	23 (12–25), 73	24 (22–26), 68	0.10

*N* number, *V*_T_ tidal volume, *mL/kg PBW* milliliters per kilogram predicted body weight, *PEEP* positive end-expiratory pressure, *P*_peak_ peak airway pressure, *P*_plat_ plateau pressure, *P*_max_ maximum airway pressure, *PaO_2_* partial pressure of oxygen in arterial blood, *FiO_2_* fraction of inspired oxygen

^a^Calculated for males as follows: 50 + 0.91 (cm of height−152.4) and for females as 45.5 + 0.91 (cm of height−152.4). Patients for which PBW could not be calculated were omitted from the analysis.

^b^Maximum airway pressure: *P*_peak_ pressure, *P*_max_ or *P*_plat_ depending on ventilator mode.

^c^Calculated as *P*_plat_ (or equivalent)−PEEP.

^d^Compliance = *V*_T_/(*P*_plat_ (or equivalent)−PEEP)

All patients were ventilated with PEEP of 5 cmH_2_O or higher, and patients with inhalation trauma received higher PEEP compared to patients without inhalation trauma [median 8 cmH_2_O (IQR 5–10) *vs* 5 cmH_2_O (IQR 5–8); *p* = 0.004; Table 2]. The median FiO_2_ did not differ between groups (Table 2). Controlled modes of ventilation were applied more frequently, with no significant differences in type of ventilator mode used for patients with or without inhalation trauma (Table 2). High-frequency ventilation was applied in 2 patients, both with inhalation trauma. *P*_max_ values <30 cmH_2_O were used in 80% of patients (Figure 1b). Median *P*_peak_ was significantly higher in inhalation trauma patients compared to patients without inhalation trauma [31 cmH_2_O (IQR 23–35) *vs* 20 cmH_2_O (IQR 17–25), *p* < 0.001]. Driving pressure did not differ between patients with and without inhalation trauma [14 cmH_2_O (IQR 11–18) *vs* 13 cmH_2_O (IQR 10–16), *p* = 0.55] and was <15 cmH_2_O in 59% of patients (Figure 1d and Table 2). Ventilatory data over the first 7 days are presented in the online supplementary appendix Figure S2.

### Clinical outcomes

**Figure 3.**
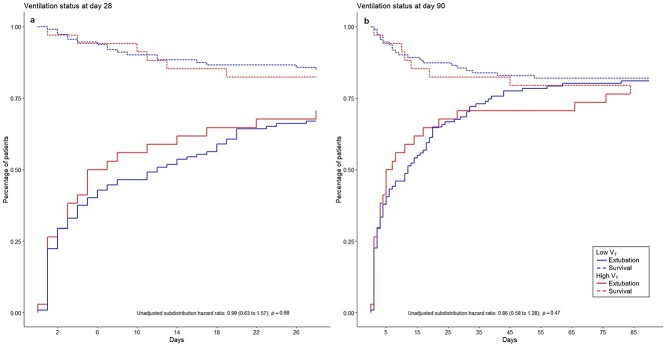
Cumulative incidence curves for ventilation status of patients ventilated with low *vs* high tidal volume size at day 28 and day 90. Sub-distribution hazard ratio: the magnitude is affected by both time to extubation and probability of death; calculated using the Cox proportional hazard model. (**a**) Ventilation status at day 28, (**b**) ventilation status at day 90. *V*_T_ tidal volume

The median number of VFD-28 was 17 (IQR 0–26) and did not differ significantly between patients ventilated with *V*_T_ ≤8 mL/kg PWB compared to *V*_T_ >8 mL/kg PWB. The subdistribution hazard ratio for extubation in patients ventilated with *V*_T_ ≤8 mL/kg PWB while considering death as a competing risk was 0.99 (0.63–1.57), *p* = 0.98 (Figure 3 and online supplementary appendix Table 3).

Patients with inhalation trauma had fewer VFD-28 compared to patients without inhalation trauma [16 (IQR 0–26) *vs* 21 (IQR 0–26)], with a subdistribution hazard ratio for extubation in inhalation trauma patients of 0.61 (0.42–0.82; *p* = 0.01), considering death before extubation as a competing risk (see online supplementary appendix Table S3 and Figure S3). Hence, patients with inhalation trauma had a 39% lower probability of being successfully liberated from MV by day 28 when compared to patients without inhalation trauma. This difference is primarily driven by the longer duration of MV (*p* = 0.02) rather than a higher mortality (*p* = 0.70, see online supplementary appendix Table S3).

Pneumonia was diagnosed significantly more often in patients with inhalation trauma (see online supplementary appendix Table S4). Most pneumonias occurred in the first week of ICU admission (53 out of 59 patients), and were diagnosed while the patient was mechanically ventilated (56 out of 59 patients). Pneumonia was microbiologically confirmed in 20 out of 38 patients with inhalation trauma *vs* 11 out of 21 patients without inhalation trauma. ARDS was reported in 28 out of 160 patients; the incidence of ARDS did not differ significantly between patients with and without inhalation trauma. The LOS in the ICU, or in hospital, and 90-day mortality did not differ between groups (see supplementary appendix Table 4).

## Discussion

This international prospective observational study investigated ventilation practices in specialized adult burn ICUs. It is one of the largest prospective cohort studies in this specific patient population over a short timeframe.

Ventilation practices in critically ill burn patients were less variable than expected as about three-quarters of the patients were ventilated with low *V*_T_, irrespective of the presence of inhalation trauma. Use of PEEP between 5 and 10 cmH_2_O and *P*_max_ values <30 cmH_2_O have also been largely adopted. This suggests that lung-protective ventilation strategies are implemented in burn patients. We found no difference in VFD-28 between patients ventilated with low compared to high *V*_T_.

The *V*_T_ in our study was marginally lower than reported *V*_T_ in nonburn patients without or at risk for ARDS [4] or ARDS patients [3]. The implementation of low *V*_T_ in the majority of patients in our study contrasts with a survey on mechanical ventilation practices amongst North American burn centers conducted in 2014. The American survey showed that ventilation practices tended to deviate from lung-protective strategies as only 26% of the respondents adhered to the ARDSNet protocol in burn patients with severe ARDS [24]. A systematic review on MV in burn patients showed a high variability in MV practices with a trend towards implementation of lung-protective settings in recent years [15]. Indeed, *V*_T_ declined from 14 mL/kg in studies performed before 2006 to ~8 mL/kg in more recent studies [15]. In our study, applied *V*_T_ did not differ between patients with or without inhalation trauma. However, patients with inhalation trauma were ventilated with significantly higher PEEP and *P*_max_. This was also seen in the systematic review on applied MV strategies in burn patients [15], where the majority of studies reported PEEP levels of up to 10 cmH_2_O. Higher PEEP levels were frequently used in studies including patients with inhalation trauma [15]. Also, studies conducted in the last decade reported the use of lower *P*_max_ values when compared to earlier studies [15]. In the present study, *P*_plat_ values were only reported for a few patients. We used *P*_max_ as a surrogate parameter for *P*_plat_ to calculate driving pressures [21]. Consequently, the calculated driving pressure could be an overestimation of the actual driving pressure. Still, driving pressures did not differ significantly between groups and were within suggested safety limits [3,25,26]. Finally, in our study, applied FiO_2_ did not differ significantly between patients with and without inhalation trauma and medians were comparable to FiO_2_ applied to critically ill patients without ARDS [4]. Currently, there are no clear recommendations on what FiO_2_ to use or what oxygen pressures in blood to aim for in burn patients. The optimal target to guide oxygen supplementation in nonburn critically ill patients also remains a subject of debate as large trials comparing liberal with conservative oxygen strategies in ventilated patients showed conflicting results [27–30]. Ventilatory data was collected at 08.00 am, provided that the patient was stable. As this was an observational study, no protocol to limit airway interventions before data collection was used. If such interventions were performed, they could have influenced the ventilatory data. For instance, *V*_T_ size could have been affected by the performance of suctioning, which may result in alveolar derecruitment [31], or by recruitment of collapsed alveoli though recruitment maneuvers [32]. Extrapolation of lung-protective ventilation strategies to the burn population is controversial as burn patients were generally excluded from benchmark ventilation studies [5,33–35]. Specific characteristics of burn patients may hamper applicability of lung-protective ventilation settings. Higher driving and plateau pressures may be required in patients with decreased pulmonary and chest wall compliance caused by circumferential abdominal and thoracic burns. We did not collect data on the percentage of total body surface area with third degree burns nor on the presence of circumferential thoracic burns requiring thoracic escharotomy. Notably, eschar formation or pulmonary edema from the injury itself or from aggressive fluid resuscitation, can increase pulmonary problems [24,36].

Also, low *V*_T_ ventilation can lead to ‘permissive hypercapnia’ [37]. Such hypercapnia may not be acceptable in burn patients who frequently require a high minute ventilation due to the markedly increased carbon dioxide production caused by the hypermetabolic response after a severe burn injury [36]. Higher *V*_T_ may be required to improve oxygenation and ventilation in burn patients [24,38,39]. The only randomized controlled trial comparing a low *V*_T_ strategy with high-frequency ventilation in adult burn patients was stopped prematurely after inclusion of 62 out of the 170 planned patients [38]. There were safety concerns as approximately one-third of the patients ventilated with low *V*_T_ failed to meet oxygenation and ventilation goals [38]. Although low *V*_T_ ventilation was applied in the majority of patients in our study, it remains uncertain whether burn patients benefit from this strategy. The lack of a relationship between *V*_T_ and patient outcome was also reported in recent cohort studies in nonburn patients [21,40]. A randomized controlled trial comparing low with intermediate *V*_T_ (i.e. 7 *vs* 9 mL/kg PBW) in nonburn ICU patients without ARDS also showed no significant difference in the number of VFD-28 or other clinical outcomes [41]. Future studies on ventilation strategies in burn patients could also target a driving pressure <15 cmH_2_O to investigate whether *V*_T_ adjusted for respiratory compliance provides better outcomes.

In our study, ARDS occurred in ~18% of patients, with no significant difference between patients with and without inhalation trauma. This is considerably higher when compared to nonburn patients [3,4]. In burn patients, the reported incidence of ARDS ranges widely [15,33,42]. Pneumonia occurred significantly more often in patients with inhalation trauma. Similar to prior studies, most cases were diagnosed within the first week post-burn [18,43]. Indeed, pneumonia is a common complication following inhalation injury and is considered an important risk factor for mortality in those patients [44].

The LAMiNAR results come with limitations. Given the lower than needed number of patients included, we simplified our analysis and focused on the impact of *V*_T_ or presence of inhalation trauma on the number of VFD-28. Still, the results of this analysis should be regarded with caution as the study was underpowered. VFD-28 was used as the main clinical outcome parameter. This composite outcome parameter does not discriminate between mortality and ventilation duration of more than 28 days [45]. To account for this limitation, we performed a competing risk analysis and reported both components of our composite outcome [45].

We did not account for potential confounders such as the severity of burn injury, severity of inhalation trauma, applied nebulizer protocols, applied fluid strategy and use of sedatives and analgesics. Timely burn wound excision and grafting could impact mechanical ventilation as it improves chest wall compliance, limits the hypermetabolic response and potentially reduces the volumes required for fluid resuscitation [46–49]. As limited data on surgical aspects of burn care were collected, we did not account for surgical procedures as possible confounding factors. Although the large number of participating specialized burn centers increases generalizability, it also means that there were only a few patients per center. We analyzed pooled data to describe current ventilation practices and did not adjust for between-center differences [50]. Also, participation bias may have occurred, as burn centers with particular interest in ventilation practices could have been more prone to participate in LAMiNAR.

## Conclusions

In this international cohort study we found that lung-protective ventilation is used in the majority of burn patients, irrespective of the presence of inhalation trauma. Use of low *V*_T_ was not associated with a reduction in VFD-28. LAMiNAR provides relevant insights into current ventilation practices in burn patients, which could serve as a baseline in future randomized trials investigating MV strategies in burn patients.

## Abbreviations

ARDS: acute respiratory distress syndrome; FiO_2_: fraction of inspired oxygen; ICU: intensive care unit; IQR: interquartile range; IRB: institutional review board; LAMiNAR: Local Assessment of MaNAgement in BuRn Patients; LIS: Lung injury scores; LOS: length of stay; MV: mechanical ventilation; PBW: predicted body weight; PEEP: positive end-expiratory pressure; *P*_max_; maximum airway pressure: *P*_peak_: peak airway pressure; *P*_plat_: plateau pressure; SAPS: Simplified Acute Physiology Score; SOFA: Sequential Organ Failure Assessment; VFD-28: ventilator-free days and alive at day 28; VILI: ventilator-induced lung injury; *V*_T_: tidal volume

## Funding

Funded by ‘Nederlandse Brandwonden Stichting’ (the Dutch Burn Association, Beverwijk, The Netherlands).

## Availability of data and material

De-identified individual participant data will be available on request from researchers, after approval of proposal by the steering committee. The data will be available by contacting the principal investigator after publication.

## Authors’ contributions

Steering committee: concept and design of the study and writing of the study protocol. National coordinators and steering committee: recruitment of participating centers. National and local coordinators: ensured the study conducted conforms to good clinical practice. Local coordinators and collaborators: patient enrollment and data collection. ASN, JMB, GJG: statistical analysis. Writing committee: data interpretation and writing of the manuscript. Steering committee and national coordinators: critical revision of the manuscript for important intellectual content. MJS: principal investigator.

## Ethics approval

The study protocol was centrally approved by the Institutional Review Board (IRB) of the Academic Medical Center at the University of Amsterdam, The Netherlands (W14_314#15.0178). All participating sites submitted the study protocol to their (local) IRB or regulatory authority and obtained ethical approval prior to initiation of the study, in compliance with the applicable local regulatory requirements.

## Consent for publication

All authors approved the manuscript and this submission.

## Competing interests

None declared.

## Supplementary Material

Supplementary_appendix_revised_tkab034
