# Supplementary material for: Ventilation practices in burn patients—an international prospective observational cohort study
Source: Burns Trauma. 2021 Dec 16;9:tkab034. doi: 10.1093/burnst/tkab034 (PMC8676707; doi:10.1093/burnst/tkab034)
Supplement: Supplementary_appendix_revised_tkab034 [file supplementary_appendix_revised_tkab034.doc]

**Supplementary Appendix**

**Ventilation Practices in Burn Patients – an International Prospective Observational Cohort Study**

LAMiNAR investigators

**TableS and figures**

**Supplement Table 1.** Patient inclusion per country

**Supplement Table 2.** List of LAMiNAR investigators and author contributions

**Supplement Table 3.** Competing risk analysis of the number of ventilator-free days and alive at day 28

Legend. Medians with interquartile ranges or counts with percentages.CI: Confidence interval. IQR: interquartile range. ml/kg PBW: milliliters per kilogram predicted body weight. VFD-28: number of ventilator-free days and alive at day 28, patients who died while receiving mechanical ventilation have zero ventilator-free days. V_T_: tidal volume.

**Supplement Table 4.** Clinical outcomes

Legend. Mann-Whitney U or Chi square test. Medians with interquartile ranges or counts with percentages.

ARDS: acute respiratory distress syndrome. ICU: intensive care unit. n/N: number of patients per group/total number of patients.

**Supplement Figure 1.** Flowchart of participating centers and patient inclusions

Legend. N: number.

**Supplement Figure 2.** Ventilation parameters in the first seven days of ventilation for patients with and without inhalation trauma

Legend: Medians with interquartile ranges. A: Tidal volume. B: Driving pressure. C: PEEP. D: Maximum airway pressure.

Abbreviations: IT: inhalation trauma; No-IT: no inhalation trauma

**Supplement Figure 3.** Cumulative incidence curves for ventilation status of patients with and without inhalation trauma at day 28 and day 90

Sub-distribution Hazard Ratio: the magnitude is affected by both: time to extubation and probability of death; calculated using the Cox proportional hazard model.

A: ventilation status at day 28. B: ventilation status at day 90

**Tables**

**Table 1.** Patient inclusion per country

|  | Country | Inclusions |
| --- | --- | --- |
| 1 | Australia | 16 |
| 2 | Belgium | 16 |
| 3 | Brazil | 22 |
| 4 | Canada | 2 |
| 5 | Denmark | 9 |
| 6 | France | 31 |
| 7 | Germany | 10 |
| 8 | Greece | 4 |
| 9 | Ireland | 1 |
| 10 | Italy | 10 |
| 11 | Netherlands | 16 |
| 12 | New Zealand | 6 |
| 13 | Norway | 1 |
| 14 | Spain | 3 |
| 15 | Sweden | 4 |
| 16 | USA | 9 |
|  | Total inclusions | 160 |

**Table 2.** List of members of the writing committee, steering committee, national coordinators and author contributions

| 1. **Writing Committee** | | **Affiliation** |
| --- | --- | --- |
| Marcus J. Schultz | | Amsterdam University Medical Centers, The Netherlands |
| Janneke Horn | | Amsterdam University Medical Centers, The Netherlands |
| Markus W. Hollmann | | Amsterdam University Medical Centers, The Netherlands |
| Benedikt Preckel | | Amsterdam University Medical Centers, The Netherlands |
| Gerie J. Glas | | Amsterdam University Medical Centers, The Netherlands |
| 1. **Steering Committee** | |  |
| Kirsten Colpaert | | Ghent University Hospital, Ghent, Belgium |
| Manu Malbrain | | Vrije Universiteit Brussel, Brussel, Belgium |
| Ary Serpa Neto | | ABC Medical School, São Paulo, Brazil |
| Karim Asehnoune | | Service d'Anesthésie Réanimation Chirurgicale, Nantes, France |
| Marcello Gamma de Abreu | | University Hospital Carl Gustav Carus, Dresden, Germany |
| Ignacio Martin-Loeches | | St James University Hospital, Dublin, Ireland |
| Paolo Pelosi | | University of Genoa, Genoa, Italy |
| Folke Sjöberg | | Linköping University Hospital, Linköping, Sweden |
| Jan M. Binnekade | | Academic Medical Center, University of Amsterdam, The Netherlands |
| Berry Cleffken | | Maasstad Hospital, Rotterdam, the Netherlands |
| Nicole P. Juffermans | | Academic Medical Center, University of Amsterdam, The Netherlands |
| Paul Knape | | Red Cross Hospital, Beverwijk, The Netherlands |
| Bert G. Loef | | Martini Hospital, Groningen, The Netherlands |
| David P. Mackie | | Red Cross Hospital, Beverwijk, The Netherlands |
| Perenlei Enkhbaatar | | University of Texas Medical Branch, Galveston, Texas, USA |
| Nadia Depetris | | Turin CTO Burn Center, Turin, Italy |
| Anders Perner | | Rigshospitalet, Copenhagen, Denmark |
| Eva Herrero | | La Paz University Hospital, Madrid, Spain |
| Lucia Cachafeiro | | La Paz University Hospital, Madrid, Spain |
| Marc Jeschke | | Ross Tilley Burn Centre, Sunnybrook Health Sciences Centre, Toronto, Canada |
| Marcus J. Schultz | | Amsterdam University Medical Centers, The Netherlands |
| Janneke Horn | | Amsterdam University Medical Centers, The Netherlands |
| Gerie J. Glas | | Amsterdam University Medical Centers, The Netherlands |
| 1. **National coordinators** | |  |
| Jeffrey Lipman | | Royal Brisbane and Women’s Hospital, Queensland University, Herston, Australia |
| Kirsten Colpaert | | Ghent University Hospital, Ghent, Belgium |
| Ary Serpa Neto | | Department of Critical Care Medicine. Hospital Israelita Albert Einstein, São Paulo, Brazil; Australian and New Zealand Intensive Care Research Centre. Monash University, Melbourne, Australia |
| Marc Jeschke | | Ross Tilley Burn Centre, Sunnybrook Health Sciences Centre, Toronto, Canada |
| Anders Perner | | Rigshospitalet, Copenhagen, Denmark |
| Matthieu Legrand | | GH St-Louis- Lariboisière, APHP, Paris, France |
| Johannes Horter | | BG Klinik Ludwigshafen, Ludwigshafen, Germany |
| Athina Lavrentieva | | Papanikoalou Hospital, Thessaloniki, Greece |
| Ignacio Martin-Loeches | | St James University Hospital, Dublin, Ireland |
| Nadia Depetris | | Turin CTO Burn Center, Turin, Italy |
| Gerie Glas | | Amsterdam University Medical Centers, The Netherlands |
| Alex Kazemi | | Middlemore Hospital, Otahuhu, Auckland, New Zealand |
| Anne Berit Guttormsen | | Haukeland University Hospital, Bergen, Norway |
| Eva Herrero | | La Paz University Hospital, Madrid, Spain |
| Lucia Cachafeiro | | La Paz University Hospital, Madrid, Spain |
| Frederik Huss | | Uppsala University Hospital, Sweden |
| Perenlei Enkhbaatar | | University of Texas Medical Branch, Galveston, Texas, USA |
| 1. **Local investigators and collaborators** | |  |
| Mark Kol | Helen Wong | Concord Repatriation General Hospital NSW, University of Sydney, Concord, Australia |
| Jeffrey Lipman | Therese Starr | Royal Brisbane and Women’s Hospital, Queensland University, Herston, Australia |
| Kirsten Colpaert | Luc De Crop | Ghent University Hospital, Ghent, Belgium |
| Manu Malbrain |  | Ziekenhuis Netwerk Antwerpen–Stuivenberg, Antwerp, Belgium |
| Wilson de Oliveira Filho |  | Hospital e Pronto Socorro 28 de Agosto, Manaus, Brazil |
| João Manoel Silva Junior | | Universidade de Sao Paulo, Sao Paulo, Brazil |
| Cintia MC Grion |  | Universidade Estadual de Londrina, Londrina, Brazil |
| Marc G. Jeschke | Marjorie Burnett | Sunnybrook Health Sciences Centre, Toronto, Canada |
| Frederik Mondrup |  | Rigshospitalet, Copenhagen, Denmark |
| Francois Ravat | Mathieu Fontaine | CHU Lyon St Luc, Lyon, France |
| Karim Asehoune | Renan Le Floch | CHU Nantes Service dánesthesie reanimation chirugicale, Nantes, France |
| Mathieu Jeanne | Morgane Bacus | Hopital Roger Salengro, CHRU Lille , Lille, France |
| Matthieu Legrand | Maïté Chaussard | Saint-Louis Hospital, Paris, France |
| Marcus Lehnhardt | Bassem Daniel Mikhail | BG University Hospital Bergmannsheil, Bochum, Germany |
| Jochen Gille |  | St George Leipzig, Leipzig, Germany |
| Johannes Horter |  | BG Klinik Ludwigshafen, Ludwigshafen, Germany |
| Athina Lavrentieva |  | Papanikoalou Hospital, Thessaloniki, Greece |
| Ignacio Martin-Loeches | Aidan Sharkey ‎ | St. James Hospital, Dublin, Ireland |
| Nadia Depetris |  | Città della Salute, Burn Center, CTO Hospital, Turin, Italy |
| Berry Cleffken | Nicole Trommel | Maasstad Hospital, Rotterdam, the Netherlands |
| Auke C. Reidinga |  | Martini Hospital, Groningen, the Netherlands |
| Paul Knape | Nadine Vieleers | Red Cross Hospital, Beverwijk, the Netherlands |
| Alex Kazemi | Anna Tilsley | Middlemore Hospital, Otahuhu, Auckland, New Zealand |
| Anne Berit Guttormsen | Henning Onarheim | Haukeland University Hospital, Bergen, Norway |
| Maria Teresa Bouza |  | Complexo Hospitalario Universitario A Coruna, a Coruna, Spain |
| Alexander Agrifoglio |  | La Paz University Hospital, Madrid, Spain |
| Frederik Huss | Filip Fredén | Uppsala University Hospital, Sweden |
| Tina Palmieri | Lynda E Painting | University of California, Davis, Sacramento, USA |
| 1. **Author contributions** | |  |
| Concept and design of the study and writing of the study protocol | | Steering committee |
| Recruitment of participating centers | | National coordinators and steering committee |
| Ensure the study conduct conform good clinical practice | | National and local coordinators |
| Patient enrollment and data collection | | Local coordinators and collaborators |
| Statistical analysis | | Ary Serpa Neto, Jan M. Binnekade, Gerie J. Glas |
| Data interpretation and writing of the manuscript | | Writing committee |
| Critical revision of the manuscript for important intellectual content | | Steering committee and national coordinators |
| Principal investigator | | Marcus J. Schultz |

**Table 3.** Competing risk analysis of the number of ventilator-free days and alive at day 28

|  | **V_T_ ≤ 8 mL/kg PBW**  **(*n* = 90)** | **V_T_ > 8 mL/kg PBW**  **(n=32)** | **Effect estimates**  **(95% CI)** | ***P* value** | **With**  **inhalation trauma**  **(*n* = 76)** | **Without**  **inhalation trauma**  **(*n* = 70)** | **Effect estimates**  **(95% CI)** | ***P* value** |
| --- | --- | --- | --- | --- | --- | --- | --- | --- |
| VFD-28 | 17.0  [0.0, 26.0] | 20.0  [0.0, 27.0] | 0.99  (0.63 to 1.57) | 0.980 | 16.0  [0.0, 26.0] | 21.0  [0.0, 26.5] | 0.61  (0.42 to 0.89) | 0.010 |
| Duration of ventilation in survivors | 5.0  [1.0, 19.0] | 6.0  [1.0, 17.0] | -1.89  (-8.95 to 5.16) | 0.598 | 11.0  [2.0, 20.0] | 3.0  [1.0, 12.0] | 7.25  (0.80 to 13.71) | 0.027 |
| Day-28 mortality, n/N (%) | 18 (20.0) | 6/32 (18.7) | 0.82  (0.32 to 2.07) | 0.672 | 13/76 (17.1) | 11/70 (15.7) | 1.17  (0.51 to 2.68) | 0.703 |

Medians with interquartile ranges or counts with percentages. CI: Confidence interval. IQR: interquartile range. mL/Kg PBW: milliliters per kilogram predicted body weight. VFD-28: number of ventilator-free days and alive at day 28, patients who died while receiving mechanical ventilation have zero ventilator-free days. V_T_: tidal volume.

**Table 4. Clinical outcomes**

|  | **All** | **With**  **inhalation trauma** | **Without**  **inhalation trauma** | ***P* value** |
| --- | --- | --- | --- | --- |
|  | **160** | **84** | **75** |  |
| **Complications, n/N** | 156/160 | 82/84 | 74/75 |  |
| ARDS, n (%) | 28 (17.6%) | 19 (23%) | 9 (12%) | 0.122 |
| Pneumonia | 59 (37.1%) | 38 (46%) | 21 (28%) | 0.037 |
| Acute kidney injury | 42 (26.4%) | 25 (30%) | 16 (22%) | 0.302 |
| Risk | 17 (11%) | 12 (15%) | 5 (7%) |  |
| Injury | 12 (7.6%) | 10 (12%) | 1 (1%) |  |
| Failure | 5 (3.1%) | 2 (3%) | 3 (4%) |  |
| Loss | 5 (3.1%) | 3 (4%) | 2 (3%) |  |
| **Length of stay in ICU, days** |  |  |  |  |
| All patients | 18 [6 – 39]  (n=150) | 19 [6 – 41]  (n=78) | 15 [5 – 39]  (n=71) | 0.35 |
| Surviving | 19 [6 – 40]  (n=116) | 19 [6 – 38]  (n=56) | 15 [5 – 40]  (n=60) | 0.30 |
| **Length of stay in hospital, days** |  |  |  |  |
| All patients | 29 [16 – 67]  (n=149) | 27 [16 – 69]  (n=77) | 39 [17 – 58]  (n=71) | 0.33 |
| Surviving | 37 [19 – 78]  (n=116) | 44 [22 – 80]  (n=55) | 28 [18–74]  (n=60) | 0.21 |
| **Mortality** | 149 | 78 | 71 |  |
| ICU – day 28, n (%) | 24 (16%) | 13 (17%) | 11 (15%) | 0.56 |
| 90 day, n (%) | 34 (23%) | 22 (28%) | 12 (17%) | 0.07 |

Mann-Whitney U or Chi square test. Medians with interquartile ranges or counts with percentages.

ARDS: acute respiratory distress syndrome. ICU: intensive care unit. n/N: number of patients per group/total number of patients.

**figures**

**Figure 1.** Flowchart of participating centers and patient inclusions


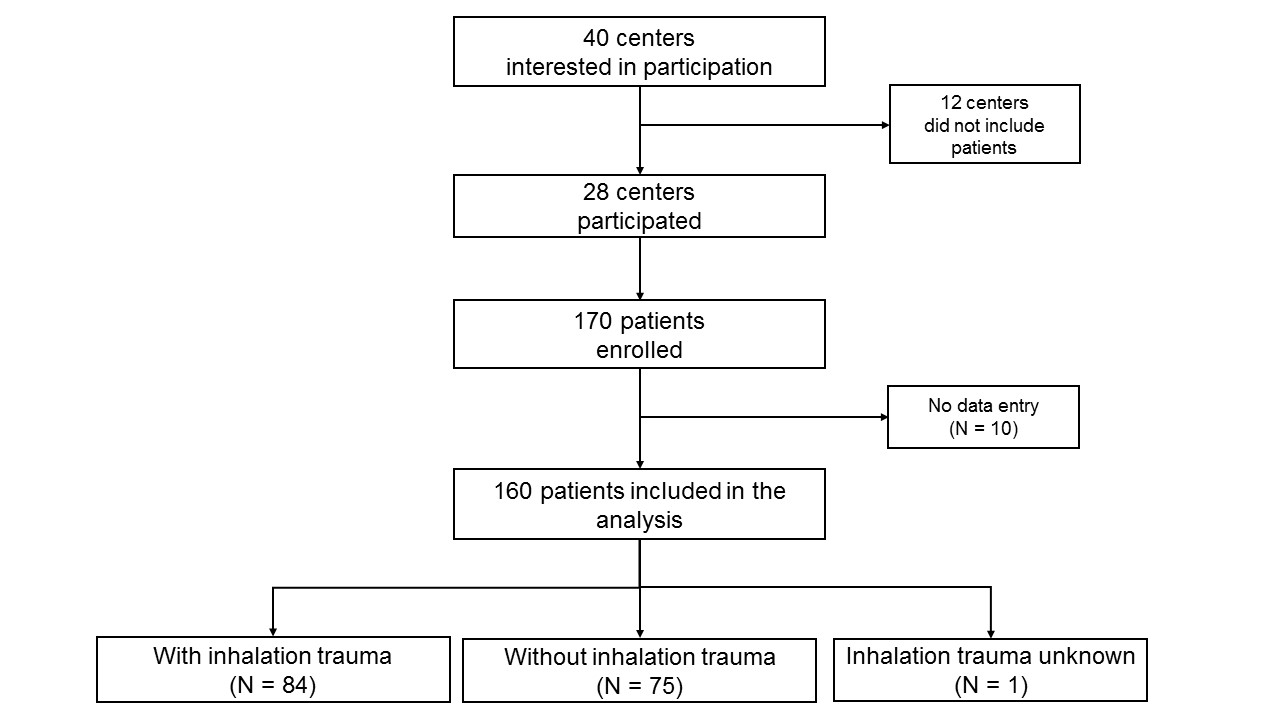


**Figure 2.** Ventilation parameters in the first seven days of ventilation in patients with and without inhalation trauma


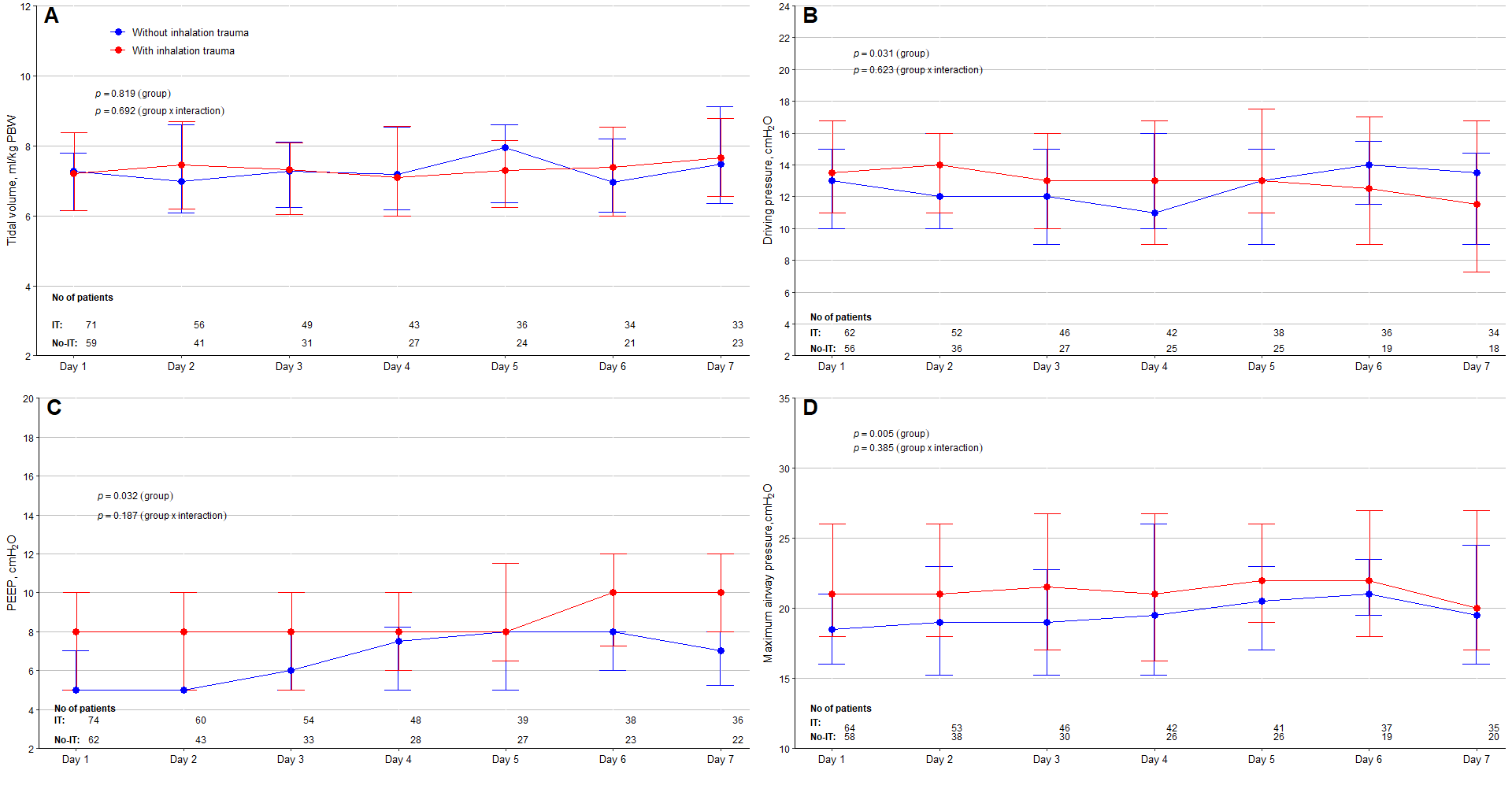


**Figure 3.** Cumulative incidence curves for ventilation status of patients with and without inhalation trauma at day 28 and day 90

**
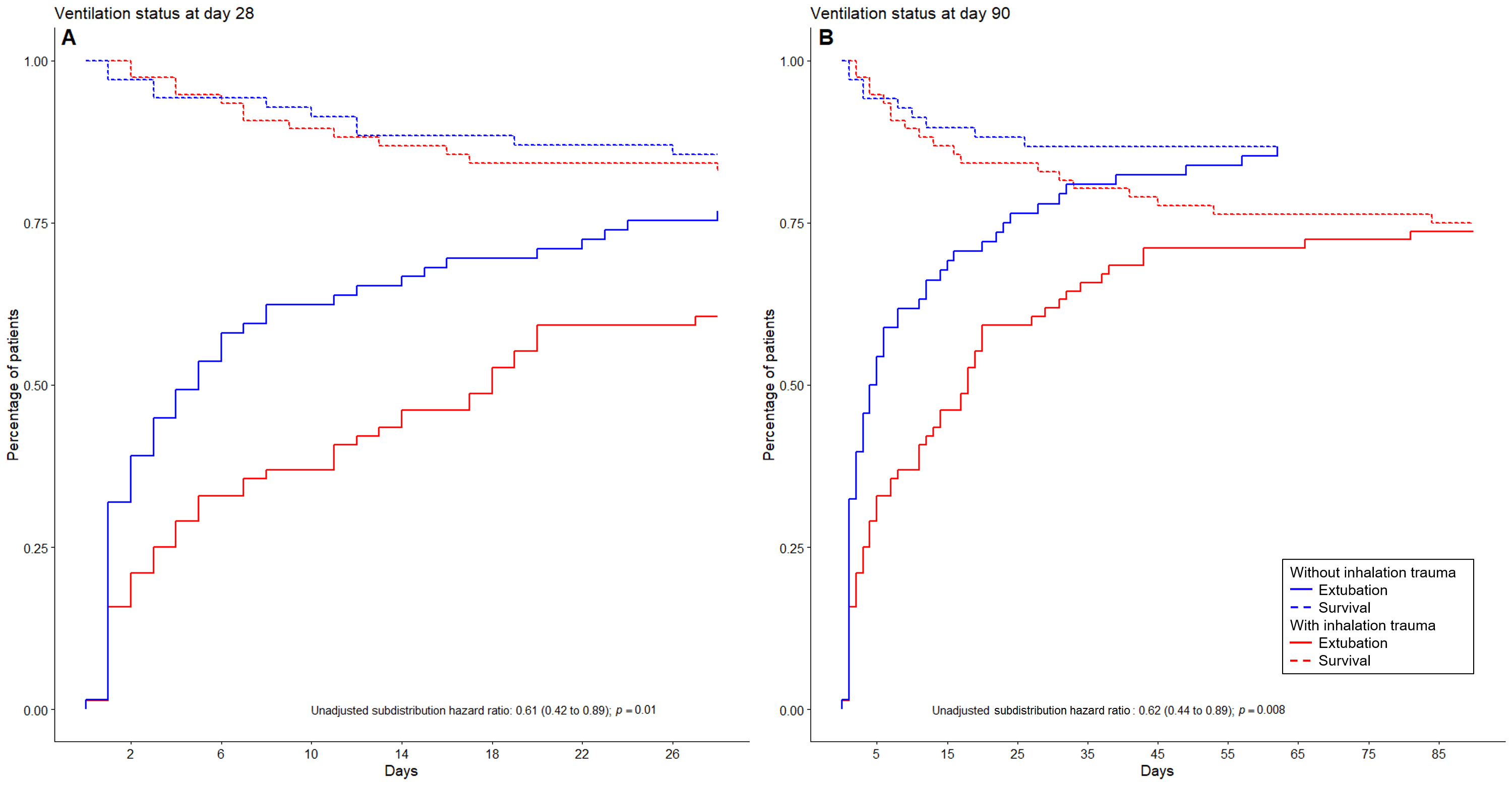
**
